# Valorization of Cork Waste in Particleboards Production with Innovative Binder

**DOI:** 10.3390/ma19030630

**Published:** 2026-02-06

**Authors:** Aleksander Hejna, Mateusz Barczewski, Jacek Andrzejewski, Adam Piasecki, Paulina Kosmela, Marek Szostak

**Affiliations:** 1 Institute of Materials Technology, Poznan University of Technology, Piotrowo 3, 61-138 Poznan, Poland; mateusz.barczewski@put.poznan.pl (M.B.); jacek.andrzejewski@put.poznan.pl (J.A.);; 2 Institute of Materials Engineering, Poznan University of Technology, Jana Pawła II 24, 61-138 Poznan, Poland; 3 Department of Polymer Technology, Gdańsk University of Technology, Narutowicza 11/12, 80-233 Gdansk, Poland

**Keywords:** cork waste, particleboards, waste management, circular economy, binder

## Abstract

Annual cork production exceeds 300,000 tons, of which over 85% is produced in Europe. Approximately 70% of cork is triturated, of which around 30% is sent to landfill and further used for energy production, which does not utilize its potential. Among potential solutions, mention should be made of cork valorization in particleboard production and of taking advantage of its exceptional properties. Herein, the study assessed the potential to manufacture novel particleboards with possible applications in the construction, building, or furniture sectors from cork waste. To enhance the innovative character and reduce environmental impact, a novel binder composed of a commonly used diisocyanate and ammonium bicarbonate was introduced. Unlike conventional resins, novel resins comprise only solid components, which makes the mixing process more straightforward. Using inexpensive inorganic salts enabled the manufacture of particleboards with increased hydrophobicity, reduced density, and enhanced thermal insulation performance, while simultaneously reducing the required amount of diisocyanate. However, these benefits were accompanied by the deterioration of mechanical performance. The obtained data suggested that by properly adjusting the materials’ composition, a compromise between density, mechanical performance, and other functionalities required by the particular applications can be achieved.

## 1. Introduction

Nowadays, besides the most broadly discussed in the public discourse, plastic or electronic waste, municipal waste is getting increasingly more attention, as this is the leading group that people are producing more and more of. One sub-stream of municipal waste is bulky waste, which mainly comprises furniture, upholstered goods, carpets, rugs, and other items [1]. Simultaneously, society’s environmental awareness is noticeably growing, as evidenced by the development of the 9R Principle: Refuse, Rethink, Reduce, Reuse, Repair, Refurbish, Remanufacture, Repurpose, Recycle, Recover [2]. These activities aim to extend the lifespans of specific products or, at least, their components, representing a step beyond the conventional linear economy, which often yields unutilized waste. Instead, the Circular Economy approach should be considered more beneficial, which involves processes that efficiently use materials, such as recycling or energy recovery. Commonly applied energy production from waste, however, is the lowest circularity level, as it generates a significant amount of by-products during various degradation processes. Nowadays, given resource shortages, an auspicious alternative that reduces operating costs and limits the harmful impact on the natural environment is the efficient recycling of bulky waste, further enabling its use as raw materials in various manufacturing processes. Following such an approach could vitally limit the use of primary raw materials and yield benefits in terms of resource efficiency. Therefore, it is vital to identify opportunities to valorize bulky waste further, enabling it to remain in a resource loop and aligning with the spirit of the Circular Economy.

Among the bulky wastes, the most common are wood, engineered wood materials, and polyurethane foams, which are widely used in the furniture industry [3]. Engineered wood materials include various products manufactured from wood or other plant-based materials, such as cork, often with a polymer resin or binder [4]. They are frequently divided by their final form or composition, but also by the type or size of applied wood/biomass particles [5]. Among the most common types are plywood, oriented strand boards, fiberboards, mass timber, veneer, and particleboards (PBs) [6]. The last group is probably the widest, as PBs may be produced from inexpensive chips, shavings, or sawdust derived from wood or other biomass [7]. Over the last few years, multiple types of biomass have been used to manufacture PBs, often yielding performance comparable to that of wood-based counterparts [8]. Some of these materials have been already introduced into industrial practice. Among them, cork should be mentioned, which is applied in the manufacturing of sound insulation or vibration damping elements, like flooring, floor underlays, or internal layers in lightweight sandwich structures [9]. In addition to its light weight, a porous structure, enhanced thermal and sound insulation, cork provides high decay resistance to final materials [10]. Moreover, cork-based materials have been reported to be more durable than other products due to their decay resistance and higher hydrophobicity, which limit moisture penetration [11]. Cork is also considered a more sustainable solution over wood, as it can be acquired without cutting down trees, and it is regenerated approximately every 10 years [12].

Cork waste originates not only from building materials, but also from wine stoppers and the packaging sector, which utilize ~60% of cork resources. However, such disposable applications generate substantial amounts of waste, which has to be efficiently utilized bearing in mind Circular Economy principles. Cork has been repeatedly analyzed in the manufacturing of various engineered wood materials potentially introduced into the building or furniture sector. Among the examples can be mentioned sandwich boards [13,14] or plywood [15,16] with cork layers, which primarily utilize layered cork. Less common examples include conventional PBs, bound with phenol-formaldehyde [17], melamine-urea-formaldehyde, and polyurethane resins [18], which use granulated cork and can be more absorptive of waste cork. This way, more sustainable solutions can be developed. A step forward could be reducing the use of adhesives, which are often synthetic and harmful [19]. An example can be found in the work of Ferrandez-Garcia et al. [20], who prepared binderless insulating boards made from Canary Island date palm and cork particles. However, in the presented case, a significant drawback of the reported solution was long processing times (even 45 min of compression molding) and a relatively high density, in most cases exceeding 700 kg/m^3^, which limited the benefits of cork’s lightweight characteristics.

The presented study aimed to meet halfway between the use of conventional binders and sustainability, and to assess the potential of manufacturing novel PBs by compression molding of cork particles together with the novel binder composition [21] consisting of one of the most commonly applied diisocyanates and ammonium bicarbonate. The advantage of the proposed solution over conventional resins lies in the physical state of binder components. Both components have been applied as solids, which significantly facilitates the proper mixing prior to PBs manufacturing, as the biomass particles do not need to be wetted by resin. The thermally induced decomposition of the applied inorganic salt led to the generation of substantial amounts of volatiles, which were entrapped in a closed mold, yielding a porous structure of the developed materials and influencing the crosslinking extent. The impact of applied formulations on the structure (density, porosity, surface roughness, and wettability), mechanical (static and dynamic) properties, thermal stability, and thermal insulation performance of the resulting PBs has been assessed. Presented data pointed to the strong relationship between PBs’ composition and performance. By proper adjustment of binder composition, the compromise between the weight, mechanical performance, and other functionalities can be found. Generally, the proposed method for bulky waste management can yield materials with performance comparable to commercially available solutions, offering novel ways to utilize them efficiently.

## 2. Materials and Methods

### 2.1. Materials

**Figure 1:**
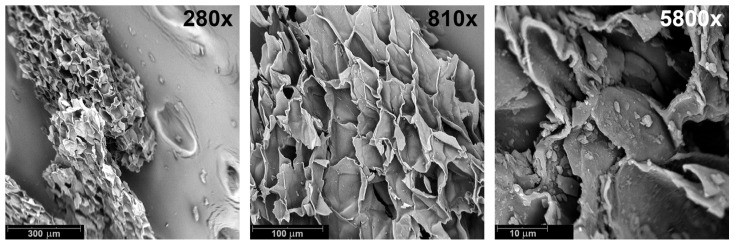
Images of applied cork powder obtained using a scanning electron microscope.

Natural cork in the form of fine granulate was supplied by CORKPOL Aleksander Kłaptocz (Ożarów Mazowiecki, Poland). The particle size ranged from 0.2 to 0.5 mm, the density equaled 0.74 g/cm^3^ (based on the gas pycnometer analysis), and the bulk density was 55–65 kg/m^3^. Figure 1 presents scanning electron microscopy (SEM) images of the applied cork material, highlighting its cellular structure, which results in a relatively low density compared to other biomass types.

The 4,4′-methylene diphenyl diisocyanate (MDI) and ammonium bicarbonate (AB) were applied as binder components during the manufacturing of the analyzed PBs. Both raw materials were acquired from Sigma-Aldrich (Poznan, Poland) and used as received.

### 2.2. Particleboards Manufacturing

Preweighted amounts of cork (200 g), MDI, and AB (total 50 g of binder composition, see Table 1), were mixed for 3 min using the planetary mixer Gerlach GL 4219 (Drzewica, Poland). Mixing time was adjusted based on preliminary works. The previously calculated amount of mixture was put in a metal mold with dimensions of 17 × 17 × 0.4 cm^3^ and shaped using compression molding on a Fontjine LabManual 300 (Rotterdam, The Netherlands) laboratory hydraulic press. The targeted density was set at 540 kg/m^3^. Table 1 provides details on applied formulations and compression molding parameters. The compression temperature was selected based on the components’ thermal stability, ensuring significant AB degradation at 100 °C, yielding the desired gas generation.

### 2.3. Characterization

Fourier transform infrared spectroscopy (FTIR) was performed using the Jasco (Hachioji, Japan) FT/IR-4600 apparatus in attenuated total reflectance (ATR) mode. FTIR analyses were carried out using 64 scans at a resolution of 2 cm^−1^ in the wavenumber range of 4000–400 cm^−1^.

Images of the PBs’ surfaces were captured using an OPTA-TECH SK (Warsaw, Poland) microscope equipped with a Meiji Techno HD2600T camera.

The roughness of the prepared PBs was assessed using an ART300 surface roughness tester from Sunpoc Co., Ltd. (Guiyang, China). The sampling length was 0.8 mm. The average profile height deviation from the mean line (R*a*) was calculated from five measurements at five different spots on the sample’s surface (25 measurements in total).

Surface wettability was studied through static water contact angle measurements using an Ossila L2004 contact angle goniometer (Sheffield, UK) equipped with a camera and Ossila Contact Angle software v3.1.2.2. Ten water contact angle (WCA) measurements were taken in random positions by placing drops of ~1 µL of distilled water onto the PB’s surface using a syringe. The average values were calculated and reported.

The analysis of the physical properties included thickness swelling and water absorption of the samples. Square-shaped specimens (50 × 50 mm^2^) were used for measurements. Specimens were thoroughly soaked in distilled water at room temperature (20–22 °C) for 2, 6, 12, and 24 h to determine water-resistance properties. The thickness and weight of the sample were measured before and immediately after it was soaked. Results, calculated according to Equations (1) and (2), are reported as percentage values before soaking:

Thickness swelling = (t_2_ − t_1_)/t_1_∙100% (1)

where t_1_ is the thickness of the test piece before immersion, mm; t_2_ is the thickness of the test piece after immersion, mm.

Water absorption = (w_2_ − w_1_)/w_1_∙100% (2)

where w_1_ is the weight of the test piece before immersion, g; w_2_ is the weight of the test piece after immersion, g.

The UV–Vis spectroscopy studies were performed to analyze the variations in appearance of water after performing thickness swelling and water absorption tests. They were conducted with the Schott UviLine 9400 (Mainz, Germany) UV-Vis spectrophotometer, operating in the range of 190–1100 nm with a resolution of 1 nm.

The PBs’ morphology was evaluated using a scanning electron microscope (SEM), Tescan MIRA3 (Brno, Czech Republic). For the SEM, a thin carbon coating of approximately 20 nm was deposited on PBs using a Jeol JEE 4B vacuum evaporator (Tokyo, Japan). The PBs’ structures were analyzed using an accelerating voltage of 5 kV. The secondary electron detector was used.

The apparent density of PBs was calculated as the ratio of weight to volume (g/cm^3^). The rectangular specimens were measured with a slide caliper with an accuracy of 0.1 mm and weighed using an electronic analytical balance with an accuracy of 0.001 g. Ten specimens have been analyzed for each composition. The obtained apparent density values have been used to calculate the compression ratio, defined as the ratio of apparent density to the targeted density of developed PBs (540 kg/m^3^).

To further investigate the structure of prepared PBs, they were analyzed using an Anton Paar Ultrapyc 5000 Foam gas pycnometer (Graz, Austria). The following measurement settings were applied: gas—nitrogen; target pressure—19.0 psi; temperature control—on; target temperature—20.0 °C; flow mode—coarse powder; cell size –10 cm^3^; preparation mode—pulse, four times. Based on the pycnometric analysis and values of apparent density, the porosity of materials has been calculated following Equation (3):

Porosity = (δ_pyc_ − δ_app_)/δ_pyc_ × 100% (3)

where δ_pyc_—density of the sample measured with a gas pycnometer, g/cm^3^; δ_app_—apparent density of the sample, g/cm^3^.

The thermal conductivity coefficient (λ) of the prepared PBs was determined using the heat flow meter HFM 446 from Netzsch (Selb, Germany). Specimens with a thickness of 4 mm were tested over the temperature range of 1.0 to 19.0 °C, with an average temperature of 10.0 °C.

The flexural strength of the PBs was measured in accordance with ASTM D790. The beam-shaped specimens with dimensions of 4 × 10 × 100 mm^3^ were measured with a slide caliper with an accuracy of 0.1 mm. The bending test was performed using a Zwick/Roell Z010 model 5101 universal testing machine (Ulm, Germany) at 25 °C and 30% relative humidity, at a constant speed of 10 mm/min. The presented data are the average of at least seven measurements from static flexural experiments. Prior to analysis, all samples were conditioned at 25 °C and 30–35% or 65% relative humidity, for at least 72 h.

The thermomechanical evaluation of PBs’ properties was performed using dynamic mechanical analysis (DMA), where viscoelastic properties were recorded during the temperature scan. For all tested PBs, the test temperature ranged from −30 °C to 150 °C, with a heating rate of 2 °C/min. A 30 µm deformation was applied at 1 Hz. The tests were conducted using the Netzsch DMA 242 E Artemis apparatus (Selb, Germany) in dual cantilever mode.

Thermogravimetric analysis (TGA) of PBs and applied components was performed using a TG F1 Libra^®^ apparatus from Netzsch (Selb, Germany). Tests were performed on samples of 8.5 ± 1.0 mg and ceramic pans. The analyses were conducted under nitrogen, in an inert atmosphere, at a flow rate of 40 mL/min. The testing range spanned from ambient temperature to 800 °C, with a heating rate of 10 °C/min.

## 3. Results

### 3.1. Chemical Rationale for the Application of MDI/AB Binder Composition

**Figure 2:**
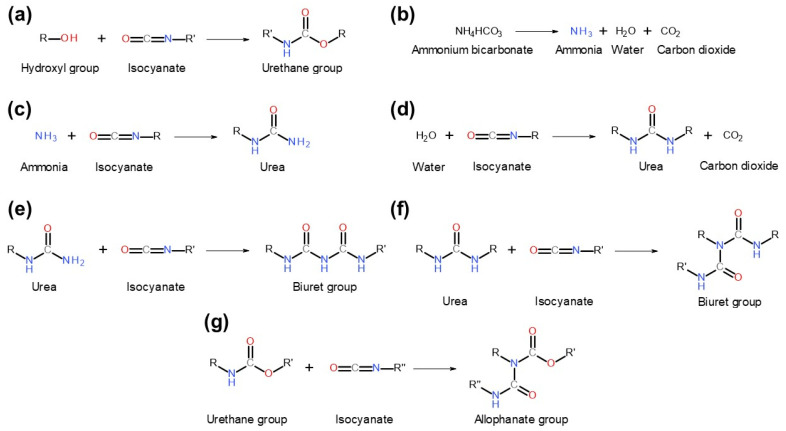
Potential chemical reactions occurring between cork powder and components of the applied binder compositions. Reactions involve the following components: (**a**) cork—hydroxyl groups and MDI—isocyanate groups; (**b**) AB—thermal decomposition; (**c**) AB—ammonia originating from thermal decomposition and MDI—isocyanate groups; (**d**) cork—residual water, AB—water originating from thermal decomposition, and MDI—isocyanate groups; (**e**) MDI—isocyanate groups and urea from reaction (**c**); (**f**) MDI—isocyanate groups and urea from reaction (**d**); (**g**) MDI—isocyanate groups and urethane from reaction (**a**).

**Figure 3:**
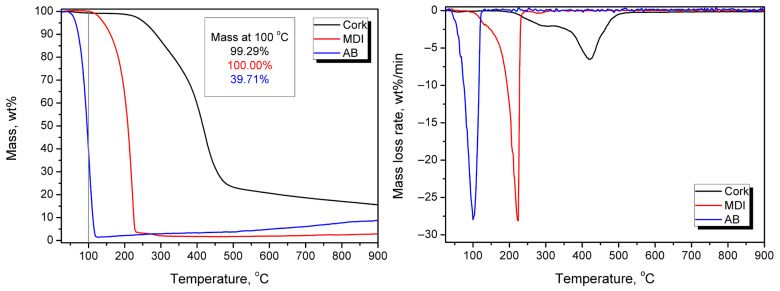
Mass loss curves and differential thermogravimetric curves for the applied raw materials.

Their composition and application followed our patent application [21] and previous work [3]. Figure 2 presents the chemical reactions that may occur between the binder components and cork, while Figure 3 presents the thermogravimetric analysis results for the applied components.

Application of MDI and other diisocyanates as binders for cork and the main component, suberin (Figure 2a), has been repeatedly reported [22,23,24]. In the presented work, the MDI:AB binder composition was applied to reduce the use of diisocyanate, which is considered harmful for human health and the environment [25]. Simultaneously, thermal decomposition of AB during sample manufacturing (Figure 2b) releases substantial amounts of gases, including ammonia, water vapor, and carbon dioxide, which may significantly affect the structure and porosity of the final PBs. Figure 3 indicates that AB decomposition is gradual and occurs at relatively low temperatures, as over 60 wt% mass loss is observed at 100 °C. Moreover, the presence of ammonia increases the pH, potentially accelerating reactions between the functional groups of cork and the MDI binder. This factor should be considered beneficial from a technological standpoint [26]. At the end, free isocyanate groups may also react with the generated ammonia and water, yielding urea moieties (Figure 2c,d) and further biuret groups (Figure 2e,f), which may enhance material crosslinking, but only when MDI particles and later urea groups are bonded with cork particles. The last possibility is the reaction of the excess of isocyanate groups with already generated urethane, yielding the allophanate moiety (Figure 2g). According to Vilar [27], in the presented case, the generation of urea during the aforementioned reaction between isocyanate and ammonia should dominate over the formation of urethane moieties (isocyanate reaction with hydroxyl groups), as the relative reactivity is significantly higher (100,000 for ammonia vs. 100 for primary hydroxyl groups). These reaction rates have been reported for uncatalyzed reactions at 25 °C, which may oversimplify such a complex system. Nevertheless, these quantitative values may provide insights into potential interactions during the development of the presented PBs.

Concluding, in the presented case, the introduction of AB, yielding ammonia generation during compression molding, most probably reduces the extent of reaction between MDI isocyanate groups and cork hydroxyls in favor of urea generation during isocyanate-ammonia reaction. This may lead to partial or complete deactivation of MDI particles towards hydroxyls and, consequently, reduced urethane generation. However, for partial deactivation (only one of the two isocyanate groups in the MDI particle reacts with ammonia), the MDI particle can still bind with cork hydroxyl, resulting in urea-functionalized cork particles, which may be further crosslinked by biuret moieties. Given the reactivity of specific groups, the increasing share of AB in the binder composition should reduce crosslink density (due to the lower isocyanate group content), while simultaneously increasing the total urea group content in the system, not necessarily bound to cork particles.

**Figure 4:**
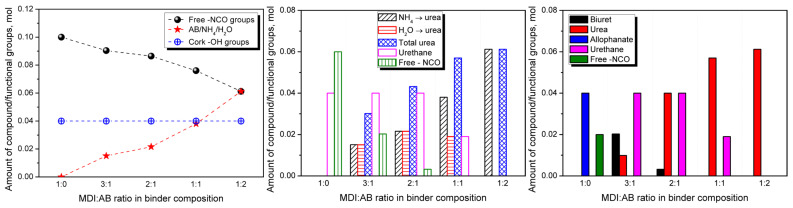
Results of theoretical calculations related to the content of particular chemical moieties within the analyzed systems.

To investigate this aspect more comprehensively, theoretical calculations of the formation of specific groups could be performed using the applied formulations, which are, however, valid only under ideal conditions. Figure 4 presents the initial content of particular moieties inside developed PBs, assuming that (i) the moisture content in applied cork is negligible, TGA results indicated only 0.7 wt% mass loss at 100 °C); (ii) the hydroxyl value of applied cork equals 0.80 mmol -OH/g, as reported by de Leon et al. [28] and aligns with 0.85 mmol -OH/g reported for bamboo fibers by Li et al. [29]; and (iii) the AB is entirely decomposed during compression molding. It can be seen that the content of isocyanate groups is inversely proportional to the AB loading and the amounts of generated NH_4_ and H_2_O, which result from the applied compositions. Their share is equal for the MDI:AB ratio of 1:2, which is attributed to the presence of two equally reactive isocyanate groups in the 4,4′-MDI particle [27]. Further, Figure 4 provides the contents of particular moieties after primary reactions of isocyanate groups with NH_4_, H_2_O (both yielding urea), and cork hydroxyls (yielding urethane), assuming that (i) the reaction rates of MDI with hydroxyl groups and water are equal [27]; and (ii) the reaction rate of isocyanate groups with ammonia is significantly higher compared to hydroxyl groups. Under these assumptions, the sequence of reactions for isocyanate groups is as follows (with literature-based reaction rates [27]): (i) reaction with ammonia yielding urea (100,000); (ii) reactions (same reaction rate) with water and cork hydroxyl groups, yielding urea and urethanes, respectively (100); (iii) reaction with urea yielding biurets (15); and (iv) reaction with urethane yielding allophanate (0.3). Therefore, after primary reactions, free isocyanate groups remain in the PBs manufactured with MDI excess in the binder composition, allowing secondary reactions that yield biurets and allophanates (see Figure 2), whose contents are shown in Figure 4. Under the assumptions above, PBs manufactured solely with MDI should be strongly crosslinked due to the noticeable excess of MDI applied (unbound isocyanate groups should still be present after secondary reactions). The introduction and rising share of AB in the binder composition should significantly increase the urea moiety content within the system. For the excess of MDI over AB, secondary reactions between urea and isocyanates should yield biuret groups. However, for equimolar MDI and AB, the MDI amount would be insufficient to undergo secondary reactions, as it would have been previously consumed by cork hydroxyls, yielding urethanes. These theoretical calculations, despite significant limitations arising from the aforementioned assumptions and simplifications, as well as the complexity of the system and processing conditions (elevated temperature and pressure), should provide important insights into the structure and performance of developed PBs.

### 3.2. Structure and Properties of Developed Particleboards

**Figure 5:**
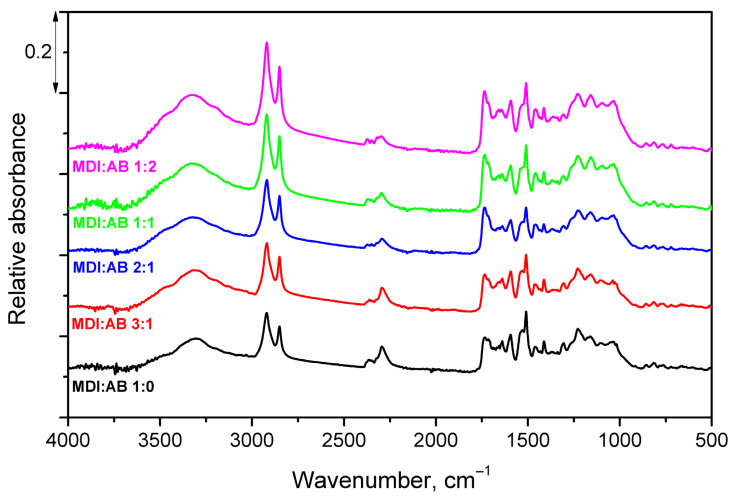
FTIR spectra of developed cork-based particleboards.

To track the variations in the chemical structure of PBs yielding from changes in binder composition, FTIR analysis has been conducted. Figure 5 presents the averaged (from at least 15 measurements) spectra of all analyzed PBs. All spectra were normalized using the intensity of the 721 cm^−1^ peak, typical for suberin. The most significant difference associated with the changes in binder composition can be noted in the range of 2240–2380 cm^−1^, where an absorption peak typical for unreacted isocyanate groups can be noted [30]. Its magnitude noticeably decreases with reduced MDI share. Except for the isocyanate-related peak, increasing intensity of broad signals in the range of 3000–3600 cm^−1^ can be observed, which is related to the stretching vibrations of N-H bonds and their higher content yielding from AB thermal decomposition during compression molding [31]. Increased magnitude of signals attributed to symmetric and asymmetric vibrations noted at 2850 and 2920 cm^−1^ can be explained by the partial decomposition of cellulose due to the increased pH originating from AB decomposition [32,33]. Changes in the region of Amide I and II bands (1500–1750 cm^−1^) have been noted; however, this region also contains multiple peaks characteristic for cork structure, e.g., C=C bonds present in suberin and lignin aliphatic groups, which impedes drawing substantial conclusions [34,35]. Nevertheless, for higher AB shares, signals at 1735, 1658 and 1640 cm^−1^, characteristic for vibrations of free carbonyl groups, hydrogen-bonded, and free ureas, were strengthened, which may confirm enhanced urea generation [36].

**Figure 6:**
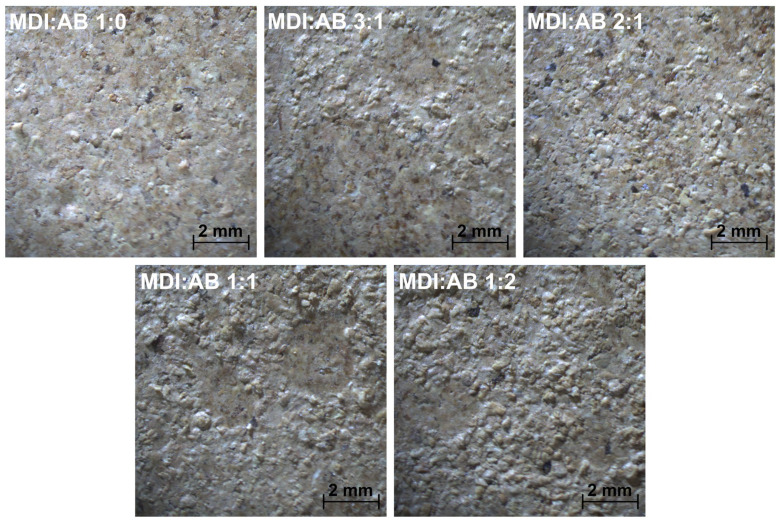
Appearance of the surface of developed particleboards (magnification 4×).

Figure 6 shows the surface appearance of the prepared PBs. It can be seen that, for the sole application of MDI as a binder, the surface is relatively flat, with only minor pinholes, which are typical of engineered wood materials like PB [37]. However, only a few peaks are observed on the surface compared to samples containing AB in the binder composition. For equivalent MDI and AB shares, or AB excess, the number of surface peaks is significantly higher, and surface roughness noticeably increases based on the visual observations. Such an effect could be attributed to the evaporation of gases generated during compression molding, as well as during sample demolding and cooling after the process.

**Figure 7:**
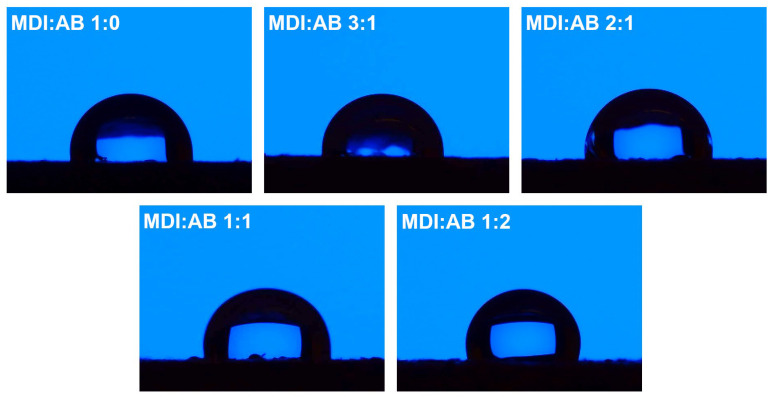
Images of water drops deposited on the surface of the developed particleboards pointing to the significant differences in surface hydrophobic/hydrophilic characteristics.

**Figure 8:**
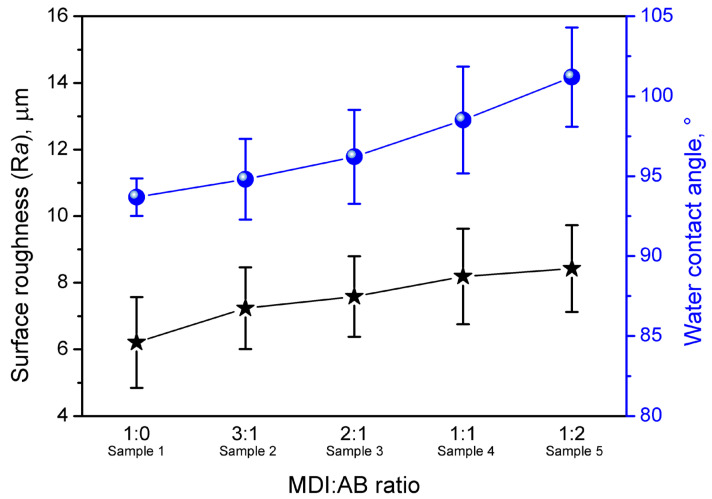
Values of surface roughness and water contact angles for the developed cork-based particleboards.

Except for the appearance of the PBs’ surface, their roughness and hydrophilicity have been affected by the variations in binder composition. Figure 7 presents the results of the WCA analysis with the goniometer, while Figure 8 presents the surface roughness values for the samples, indicating significant differences between the compositions. For all of the analyzed PBs, WCA values point to hydrophobic characteristics of the surface, which could be attributed to the chemical composition of cork; more precisely, the high content of suberin [38]. The results presented indicate that surface hydrophobicity can be enhanced by adjusting the MDI:AB binder composition, specifically by increasing AB loading. Such an effect has been noted despite the higher hydrophilicity of urea groups compared to urethanes generated during MDI reaction with hydroxyl moieties present in cork structure (Figure 2). Therefore, values of WCA obtained from goniometer measurements were probably impacted by the increasing surface roughness, potentially induced by the enhanced gas generation resulting from AB thermal decomposition during compression molding. Observed changes in surface characteristics should be considered auspicious for the potential application of the developed PBs, as higher hydrophobicity yields enhanced resistance to moisture and atmospheric conditions [39].

**Figure 9:**
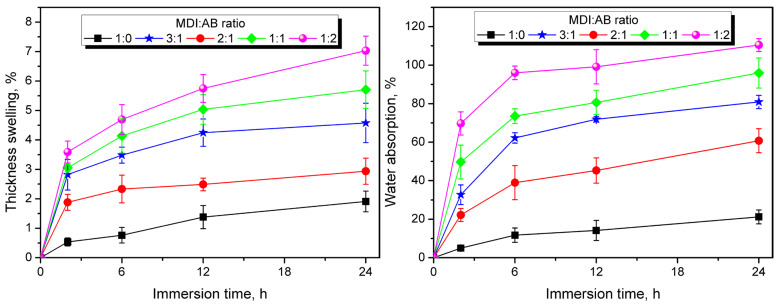
Results of thickness swelling (**left**) and water absorption (**right**) tests performed for the developed particleboards.

In addition to the water resistance of PBs’ surfaces, interactions throughout the material’s volume are critical. Therefore, Figure 9 presents the results of the water absorption and thickness swelling tests, indicating variations arising from the binder composition. It can be seen that the water absorption and thickness swelling curves exhibit a course typical of PBs, with an initial rapid increase and a tendency to reach equilibrium with prolonged exposure [40,41]. Considering the values of these parameters, they have clearly increased with AB content in binder composition. Such an effect contradicts the results of the surface wetting analysis presented above. However, it aligns with the reduced crosslink density suggested by theoretical calculations, the increasing urea content associated with variations in binder composition and their higher hydrophilicity compared to urethanes. Moreover, further reported decrease in apparent density observed in the SEM images and in the density analysis, as well as higher porosity contributed to the easier penetration of samples with water and higher absorption. Lower density implies higher void content, while the decreased crosslink density leads to higher structural mobility. Together with the hydrophilicity of urea moieties, these factors facilitate the penetration of water into the PBs structure via capillary absorption, leading to increasing thickness-swelling values. The unfavorable impact of introducing hydrophilic components has been confirmed by Ferrandez-Garcia et al. [20], who used Canary Island palm particles in place of cork, resulting in a 10-fold increase in thickness swelling.

Nevertheless, the obtained thickness-swelling values were significantly lower than the requirements for non-structural PBs, as stated in the EN 312 standard [42] for P3 type—17% after 24 h. Moreover, they were lower than those for cork-based PBs manufactured with phenol–formaldehyde adhesive, which, depending on the density (from 550 to 725 kg/m^3^), ranged from 8.31 to 9.35% after 24 h immersion [17]. On the other hand, Ferrandez-Garcia et al. [20] reported values of 1.61–1.62% for the urea-formaldehyde resin-bound cork PBs, which have been matched only by the PB prepared solely with MDI as a binder.

**Figure 10:**
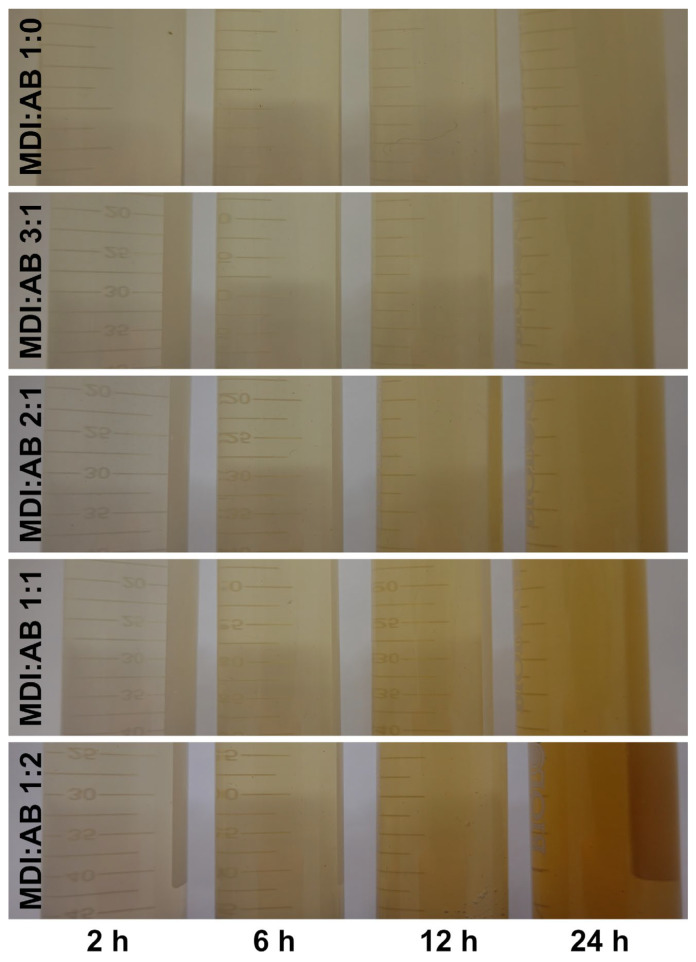
Appearance of the residual water after immersion of developed PBs.

**Figure 11:**
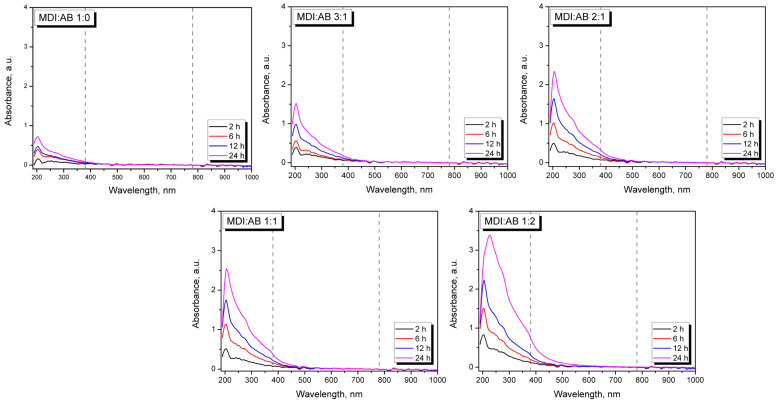
UV-VIS spectra plotted for the residual water after immersion of developed PBs. Dashed lines separate ultraviolet (UV), visible (VIS) and infrared (IR) regions on the spectra.

Notably, after 24 h of immersion in water, significant differences in the color of the residual water have been observed, as shown in Figure 10, which point to the extraction of structural components of cork-based PBs. For a more detailed analysis, residual water was analyzed by UV–Vis spectroscopy, and the resulting spectra are shown in Figure 11. Visual observations clearly indicate the differences arising from the binder composition. For the sole application of MDI, the resulting water was only slightly colored, as confirmed by very low absorbance values that increased only in the 380–420 nm range, characteristic of yellow shades. Increasing the AB content in the binder composition intensified the color of the water after PBs immersion, as confirmed by increased absorbance values, however, only up to 500 nm, in the region characteristic of yellow and orange colors.

The spectral pattern in the UV region (below 380 nm) confirms the potential extraction of particular components of PBs. For almost all analyzed samples, the maximum absorbance in the UV region was observed at 200–205 nm, indicating the presence of compounds with isolated double bonds, such as dienes, or simple benzene rings [43]. The absorbance peak slightly shifts towards longer wavelengths with increasing immersion time and AB loading, but the most significant shift is observed for the 24 h immersion of the MDI:AB 1:2 sample, suggesting extraction of higher-molecular-weight or more complex compounds containing polar groups, such as phenols [44]. Such an interpretation can also be confirmed by the increasing absorbance of the shoulder peak, with a maximum in the range of 270–280 nm, typical of substituted benzene rings [45]. Observed changes in the appearance of water after PBs immersion points to a potential direction for further work on the development of novel PBs and other engineered wood materials, related to their potential impact on the environment and human health. However, the detailed analysis of extracted components is beyond the scope of the presented study.

**Figure 12:**
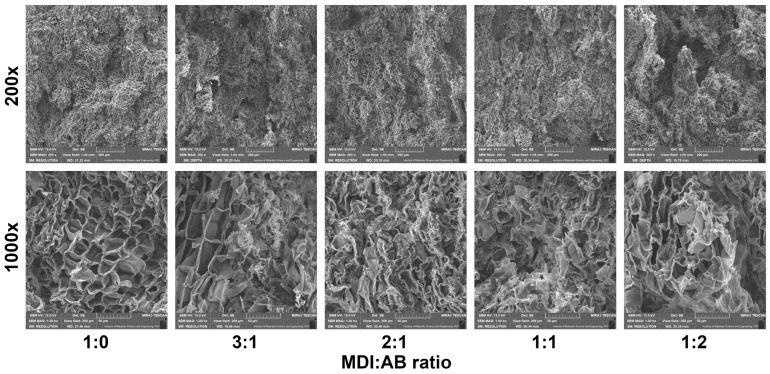
SEM images of cross-sections of developed cork-based materials in different magnifications.

**Figure 13:**
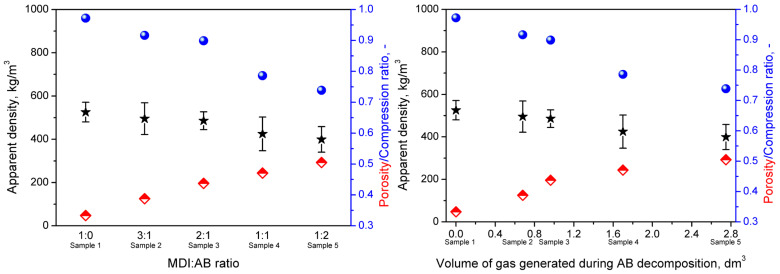
The impact of MDI:AB ratio in binder composition (**left**) and theoretical volume of gas generated during AB decomposition (**right**) on the apparent density of developed particleboards and compression ratio.

Figure 12 presents SEM images of the morphology of the developed PBs, which reveal their cellular structure and confirm the relatively low apparent density and high porosity values reported in Figure 13. Notably, variations in binder composition led to significant changes in cell shape, attributed to additional gas generation from the thermal decomposition of AB (see Figure 2b). For the sole application of MDI, gas generation can result only from isocyanate reactions with residual moisture in cork. Therefore, it can be seen that the shape of cells in Sample 1 (MDI:AB 1:0) is similar to the cork raw material (Figure 1). Some differences can be obviously attributed to the compression molding procedure, which assumes material densification, potentially destructive to cork cellular structure. Nevertheless, a relatively low amount of generated gas yielded a high compression ratio of 0.972, indicating that the targeted density has been almost achieved.

On the other hand, AB introduction noticeably increased gas generation (at 100 °C, 3 moles of gas per mol of AB, see Figure 2b). Even considering the high ammonia reactivity towards isocyanate groups, the gas amount gradually increased with the AB content in the binder composition, as reflected in the PBs’ morphology, which yielded higher open porosity. The theoretical amount of gas generated for each sample (based on mass input and stoichiometry) is shown in Figure 13 and plotted against the apparent density and compression ratio. A very high value of Pearson correlation coefficient of 0.980 has been noted for the relationship between apparent density and the calculated amount of AB-originated gas, which suggests that it could be possible to efficiently engineer the final PBs’ density by the adjustment of MDI:AB binder composition. For the AB excess in the binder composition (Sample 5), the initial cork cellular structure has been hardly preserved due to the additional pressure induced by the higher gas amount in the closed mold cavity.

**Figure 14:**
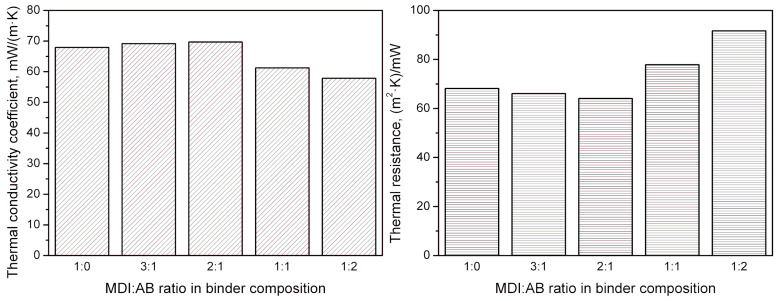
Values of thermal conductivity coefficient (**left**) and thermal resistance (**right**) as a function of MDI:AB ratio in binder composition for developed particleboards.

The aforementioned variations in the structure of developed PBs typically show a noticeable impact on their performance. Considering the potential applications in the building sector, thermal insulation performance should be mentioned among the critical features [46,47,48]. Therefore, Figure 14 provides insights into the thermal conductivity coefficients (λ) and the thermal resistance of the prepared cork-based PBs. It can be seen that using solely MDI or MDI excess in combination with AB yielded relatively similar values of λ coefficients in the range of 67.9–69.7 mW/(m∙K), which are lower than repeatedly reported for medium-density fiberboards or other particleboards with similar density [49,50]. Lower values in the range of 40–50 mW/(m∙K) have also been reported, but for the materials characterized with the density of 90–240 kg/m^3^, which is significantly lower than in the presented work [51,52]. However, equimolar shares of MDI and AB, or AB excess, resulted in beneficial changes in thermal insulation performance and reduction in λ coefficient to 61.3 and 57.9 mW/(m∙K), respectively. This effect can be attributed to the noticeable decrease in apparent density, as confirmed by the high Pearson correlation coefficient of 0.918, indicating a strong relationship between the two parameters. A significantly higher amount of gas generated during AB thermal decomposition yielded a porous structure, thereby increasing the gas share within the analyzed volume of material. Such an effect is very beneficial for thermal insulation materials, as reported λ coefficients of carbon dioxide and air are 15.3 and 24.9 mW/(m∙K), respectively, which is noticeably lower than for solid materials [53,54]. Moreover, the SEM images in Figure 12 suggest that the structure of the developed PBs contains a portion of closed cells, which may further enhance insulation performance by slowing the carbon dioxide-to-air exchange rate [55].

**Figure 15:**
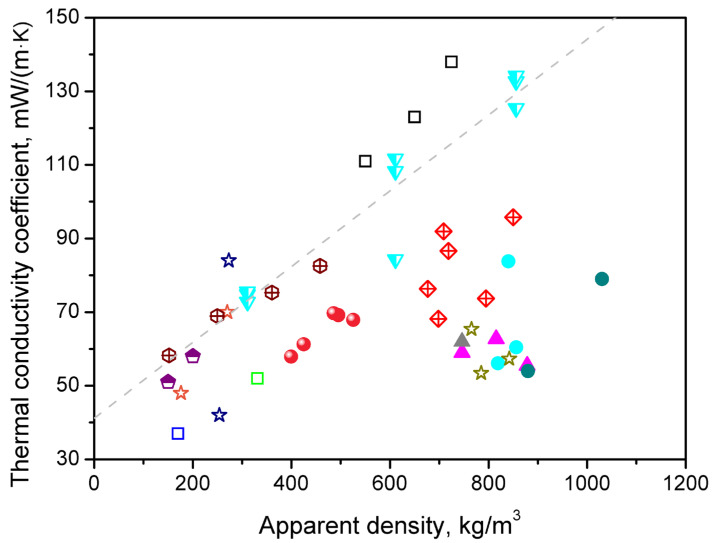
Summary of the literature reports on the thermal conductivity of various non-conventional PBs. Dashed line was fitted to the introduced data. The results for following raw materials have been provided: cork—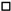 [17], 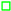 [20], 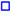 [56]; cork + Canary palm—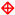 [20]; Canary palm—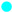 [57], 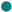 [63]; date palm—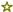 [57], 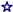 [58], 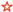 [59]; Washingtonia palm—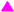 [57], 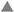 [64]; kenaf fiber—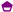 [60]; cotton stalk—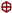 [61]; and durian peel + coconut coir—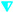 [62]. The results from this work have been marked with symbol 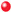.

Considering cork-based PBs described in the literature, Ferrandez-Garcia et al. [20] reported a λ coefficient of 52 mW/(m∙K) for the density of ~331 kg/m^3^ and values ranging from 68 to 96 mW/(m∙K) for PBs manufactured from a combination of cork and Canary Island palm characterized with the density of 676–850 kg/m^3^. On the other hand, Lakreb et al. [17] manufactured cork-based PBs with densities of 550, 650, and 725 kg/m^3^, and reported λ coefficient values of 111, 123, and 138 mW/(m∙K), respectively. For a more detailed comparison, Figure 15 summarizes the literature reports dealing with the thermal conductivity of various non-conventional PBs [17,20,56,57,58,59,60,61,62,63,64]. A clear trend between apparent density and λ coefficient can also be noted for other works, and deviations from the fitted line may be attributed to the type of applied binder.

**Figure 16:**
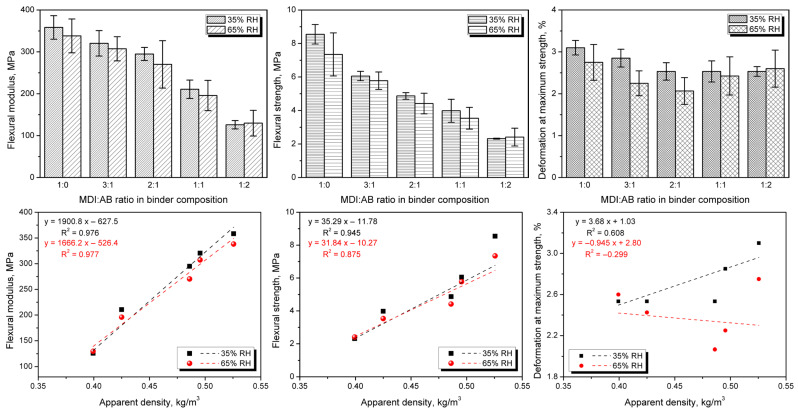
The impact of MDI:AB ratio in binder composition and apparent density on flexural modulus (**left**), flexural strength (**middle**), and deformation at maximum strength (**right**) for developed cork-based particleboards.

Despite the beneficial impact of AB application on thermal insulation performance, there is another side to this coin—mechanical performance, which, depending on the final application, might be even more critical. Therefore, to assess the impact of formulation adjustments, Figure 16 presents the flexural performance of the developed PBs after conditioning at different relative humidity. A significant deterioration in modulus and strength has been observed with increasing AB loading. Such an effect could be attributed to the enhanced gas generation and lower apparent density, which have been repeatedly reported to affect the mechanical performance of engineered wood materials [65,66]. A strong correlation between apparent density and flexural performance has also been confirmed by an almost proportional relationship between elastic modulus and strength. Considering the impact of relative humidity, deterioration of the mechanical properties has been noted, except for the highest AB content in binder composition, where similar values of modulus and strength have been noted. Nevertheless, for all of the analyzed PBs, the differences are within the values of standard deviation.

**Figure 17:**
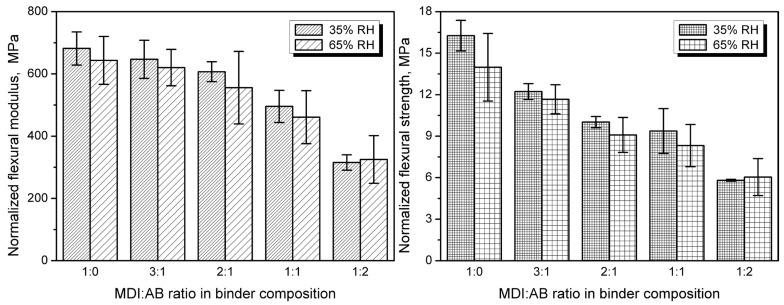
Normalized values of flexural modulus and flexural strength for developed cork-based particleboards.

On the other hand, for cellular materials, mechanical performance is strongly driven by the apparent density, which quantifies the actual share of solid material in a given volume [67,68,69]. Figure 17 shows the normalized flexural modulus and strength. Such an approach is typical for evaluating engineered wood materials, which may differ significantly in apparent density [70,71]. The resulting information should provide further insights into the impact of binder composition on interparticle adhesion in the developed PBs.

It can be seen that, despite normalization, the flexural properties have deteriorated with increasing AB share, indicating weakened interparticle adhesion. In terms of modulus, the most significant effect was observed for the AB excess, which could be attributed to the lack of urethane bonds within the material. As mentioned above, the increase in the total urea content has not implicated the stiffening of the PBs, as these moieties could be only deposited on the cork particles. On the other hand, flexural strength was strongly reduced, even with the AB at the lowest 25% share, confirming the assumption of reduced crosslink density in the PBs caused by the allophanate and biuret presence, despite the more pronounced urea generation.

Nevertheless, obtained results hardly differ compared to other works in the literature on cork-based PBs, which has been summarized in Table 2. Inferior properties have been reported for cotton stalk-based PBs [61], while relatively similar for kenaf fiber [60] and combination of cork with Canary palm [20]. Notably, cork-based PBs manufactured by Lakreb et al. [17] and Cha et al. [72] contained conventional phenol–formaldehyde and liquid polymeric MDI adhesives, respectively, which are commonly applied in industrial practice.

**Figure 18:**
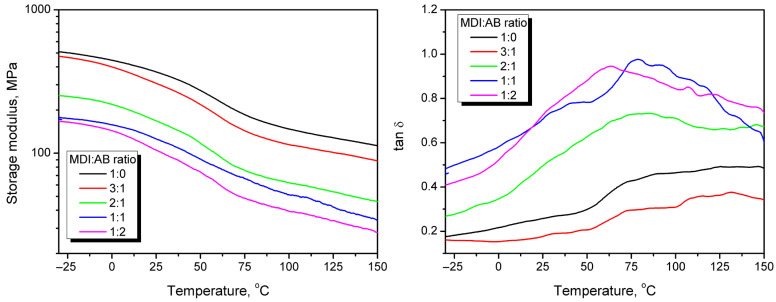
Temperature plots of storage modulus (**left**) and loss tangent (**right**) obtained during DMA of cork-based particleboards.

The results of static bending tests, particularly the conclusions related to the crosslinking degree and PBs’ stiffness, have been confirmed by the results of DMA presented in Figure 18. The storage modulus of the developed PBs decreases almost proportionally with increasing temperature, which has been attributed to the loosening of the structure of the main cork components, such as suberin, similar to that observed for various wood types [73]. Due to the specific binder used, no glass transition of the polymeric resin can be distinguished, as in the case of multiple engineered wood materials [74,75]. However, the relaxation phenomenon can be observed in both modulus and loss tangent plots, which aligns with previous works on the dynamic mechanical analysis of cork [76,77]. The increasing AB share in the binder composition reduced the storage modulus and led to a higher magnitude of tan δ peaks. Such changes indicate inferior interfacial interactions and an enhanced structural ability to dissipate energy through molecular motions, confirming the reduced crosslink density suggested by theoretical calculations (Figure 4), as well as an increased number of pores entrapped in the final PB structure as a result of the release of gaseous products of AB decomposition.

**Figure 19:**
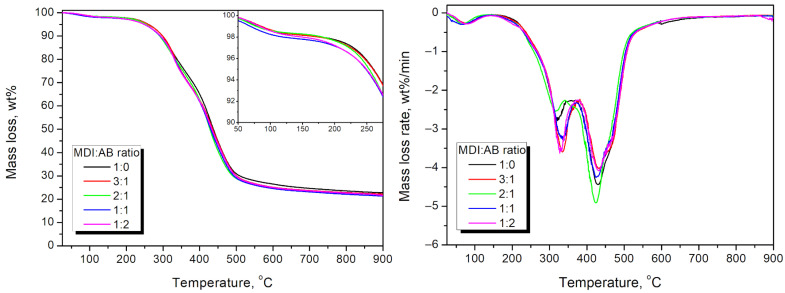
Mass loss curves (**left**) and differential thermogravimetric curves (**right**) for the developed cork-based particleboards.

To further investigate the performance of the developed cork-based PBs and the impact of the applied binder composition, TGA was conducted, and the results are presented in Figure 19. It can be seen that the MDI:AB ratio had little effect on the thermal degradation profile, since all of the materials showed a similar course of degradation as applied cork. They exhibited less than 2 wt% mass loss at 100 °C, indicative of moisture evaporation. Such an effect points to the aforementioned hydrophobic character attributed to the cork chemical composition, since wood-based particleboards typically show higher mass loss at this stage [78,79]. PBs manufactured with an MDI excess over AB showed slightly higher thermal stability, which may be associated with differences in the degradation profiles of urethane, urea, biuret, and allophanate moieties.

## 4. Conclusions

The study presented herein yielded vital insights into the use of cork waste as a raw material for novel PBs with potential applications in the construction and building sectors. The materials were produced using a standard, relatively simple compression-molding process. An innovative composition of widely applied methylene diphenyl diisocyanate and ammonium bicarbonate was introduced as a binder for cork particles. A simple yet environmentally favorable change has altered the mechanism of PBs’ crosslinking, leading to additional chemical interactions and enhanced gas generation. The impact of these reactions on the surface properties, morphology, mechanical, thermal, and insulation performance of manufactured PBs has been assessed. The introduction of ammonium bicarbonate led to significant changes in the internal and surface structure and properties. Surface roughness increased by ~33%, accompanied by a shift in the water contact angle from 93.7 to 101.2°, indicating a significant change towards hydrophobicity. Such an effect should be considered very auspicious, as it typically reduces moisture absorption and limits water penetration into the material. A substantial increase in gas generation also reduced apparent density from 526 to 399 kg/m^3^ and the compression ratio from 0.97 to 0.74, yielding a higher structural porosity and a beneficial decrease in the thermal conductivity coefficient from 67.9 to 57.9 mW/(m·K). Nevertheless, higher porosity caused deterioration in mechanical performance, with reductions in flexural modulus and strength from 358 and 8.6 MPa to 126 and 2.3 MPa, respectively. Such an effect was also associated with a reduced share of urethane moieties within the system and limited crosslinking possibilities. In conclusion, the obtained data indicate that by adjusting the MDI/AB binder composition, the performance of the developed PBs can be engineered to achieve a proper balance between mechanical performance and insulation properties for the particular products, e.g., to replace fiberboard in less demanding applications such as masking or finishing materials.

## Figures and Tables

**Table 1:** Compositions applied during particleboards manufacturing and compression molding parameters.

Sample	Content, wt%			Temperature, °C	Time, min	Pressure, bar
	Cork	MDI	AB			
1	80	20.0	0.0	100	2	20
2	80	15.0	5.0	100	2	20
3	80	13.3	6.7	100	2	20
4	80	10.0	10.0	100	2	20
5	80	6.7	13.3	100	2	20

**Table 2:** Summary of the literature reports on the mechanical performance of various non-conventional PBs.

Material	Density, kg/m^3^	MOE, MPa	MOR, MPa	Norm. MOE, MPa	Norm. MOR, MPa	Ref.
Cork	402	25.4	1.20	63.1	2.98	[72]
	550	58.5	0.71	106.4	1.29	[17]
	650	157.7	1.02	242.6	1.57	
	725	143.6	1.52	198.1	2.10	
	331	48.5	1.92	146.5	5.80	[20]
Cork + Canary palm 50:50	677	281.2	4.71	415.4	6.96	
	719	301.0	4.75	418.6	6.61	
	709	334.7	5.70	472.1	8.04	
	698	247.5	4.62	354.6	6.62	
	795	742.6	7.41	934.1	9.32	
	850	792.1	8.86	931.9	10.42	
Canary palm	856	1567.2	13.97	1830.8	16.32	[57]
	840	2018.6	19.85	2403.1	23.63	
	819	1373.6	12.68	1677.1	15.48	
Washingtonia palm	878	1526.4	16.95	1738.5	19.31	
	815	1208.2	12.40	1482.5	15.21	
	746	663.0	7.38	888.7	9.89	
Date palm	842	1263.1	13.51	1500.1	16.05	
	765	988.1	10.76	1291.7	14.07	
	785	695.0	7.85	885.3	10.00	
Kenaf fiber	107	48.4	0.29	452.3	2.71	[60]
	118	32.3	0.20	273.7	1.69	
	130	81.6	0.29	627.7	2.23	
	175	209.7	0.67	1198.3	3.83	
	202	322.6	1.59	1597.0	7.87	
	264	532.2	2.14	2015.9	8.11	
	278	612.9	2.33	2204.7	8.38	
Cotton stalk	350	27.0	0.14	77.1	0.40	[61]
	350	54.3	0.32	155.1	0.91	
	350	72.5	0.53	207.1	1.51	
	350	57.7	0.42	164.9	1.20	
	350	72.5	0.53	207.1	1.51	
	350	80.9	0.58	231.1	1.66	
Durian peel and coconut coir 90:10	311	83.9	0.70	269.6	2.25	[62]
	611	617.9	8.74	1011.3	14.30	
	856	1417.3	18.10	1655.7	21.14	
	311	146.4	2.93	470.7	9.42	
	611	791.2	15.39	1294.9	25.19	
	856	2239.2	36.16	2615.9	42.24	
	311	210.7	5.16	677.6	16.59	
	611	1152.4	27.01	1886.1	44.21	
	856	2126.8	43.20	2484.6	50.47	

## Data Availability

The original contributions presented in this study are included in the article. Further inquiries can be directed to the corresponding author.

## Abbreviations

The following abbreviations are used in this manuscript:

λ	Thermal conductivity coefficient
AB	Ammonium bicarbonate
ATR	Attenuated total reflectance
DMA	Dynamic mechanical analysis
FTIR	Fourier transform infrared spectroscopy
MDI	4,4′-Methylene diphenyl diisocyanate
PBs	Particleboards
SEM	Scanning electron microscopy
TGA	Thermogravimetric analysis
WCA	Water contact angle
